# BrainNet: an automated approach for brain stress prediction utilizing electrodermal activity signal with XLNet model

**DOI:** 10.3389/fncom.2024.1482994

**Published:** 2024-10-24

**Authors:** Liao Xuanzhi, Abeer Hakeem, Linda Mohaisen, Muhammad Umer, Muhammad Attique Khan, Shrooq Alsenan, Shtwai Alsubai, Nisreen Innab

**Affiliations:** ^1^College of Electronic and Information Engineering, Beibu Gulf University, Qinzhou, China; ^2^Department of Information Technology, Faculty of Computing and Information Technology, King Abdulaziz University, Jeddah, Saudi Arabia; ^3^Department of Computer Science & Information Technology, The Islamia University of Bahawalpur, Bahawalpur, Pakistan; ^4^Department of AI, College of Computer Engineering and Science, Prince Mohammad Bin Fahd University, Al Khobar, Saudi Arabia; ^5^Information Systems Department, College of Computer and Information Sciences, Princess Nourah bint Abdulrahman University, Riyadh, Saudi Arabia; ^6^Department of Computer Science, College of Computer Engineering and Sciences, Prince Sattam bin Abdulaziz University, Al-Kharj, Saudi Arabia; ^7^Department of Computer Science and Information Systems, College of Applied Sciences, AlMaarefa University, Diriyah, Saudi Arabia

**Keywords:** brain stress monitoring, XLNet, smart healthcare, EEG monitoring, artificial intelligence, Swell, WESAD

## Abstract

Brain stress monitoring has emerged as a critical research area for understanding and managing stress and neurological health issues. This burgeoning field aims to provide accurate information and prediction about individuals' stress levels by analyzing behavioral data and physiological signals. To address this emerging problem, this research study proposes an innovative approach that uses an attention mechanism-based XLNet model (called BrainNet) for continuous stress monitoring and stress level prediction. The proposed model analyzes streams of brain data, including behavioral and physiological signal patterns using Swell and WESAD datasets. Testing on the Swell multi-class dataset, the model achieves an impressive accuracy of 95.76%. Furthermore, when evaluated on the WESAD dataset, it demonstrates even higher accuracy, reaching 98.32%. When applied to the binary classification of stress and no stress using the Swell dataset, the model achieves an outstanding accuracy of 97.19%. Comparative analysis with other previously published research studies underscores the superior performance of the proposed approach. In addition, cross-validation confirms the significance, efficacy, and robustness of the model in brain stress level prediction and aligns with the goals of smart diagnostics for understanding neurological behaviors.

## 1 Introduction

Having outlined the goals and objectives of occupational health psychology, it is possible to focus on stressing that stress, an essential factor that affects both health and wellbeing, is still one of the main concerns of the modern world (Adochiei et al., 2019). As noted, stress refers to the broad Universal experience of organismic transactions defined as reactions to internal or external stimuli, including benefit stress that enables individuals to adapt to new situations or demanding pressures or negative stress or pressures, which have adverse effects on the organism (Zalabarria et al., 2020). This inherent mechanism works as the body's way of handling bad conditions, trying to bring balance to the body at all times (Sharma, 2018). For example, stress-related problems are one of the most common health problems and form a large proportion of health demands in most European countries and the United States, demonstrating the extent of their effects on the health of nations (Akmandor and Jha, 2017).

The first level of stress may develop when an organism is faced with a stimulus or event which is referred to as stressors (Sharma, 2018). These can be described as being the following three main types, in which two subgroups can be distinguished based on the nature of the stressors: internal and external stress variables/stressors, which can be psychological and physiological. These are some of the reasons that were classified as causes of psychological stress; these include debt, bereavement, joblessness, and studies. However, positives include infections, climate, extremes, and lack of proper rest as stressors. If the body detects a stress-causing circumstance, the body will trigger short- or long-term stress responses. This is governed by the hypothalamus, which is a very important part of the brain when it comes to stress. Gluactivates the pituitary gland to release cortisol into the adrenal gland. In addition to these functions, cortisol helps regulate blood glucose levels and bring the body to its normal functioning. However, the adrenal medulla, which is part of the ANS stimulated by the hypothalamus, releases fast stress responses. This produces adrenaline that triggers the fight or flight response and starts the sympathetic division. The stressor is no longer present, and the parasympathetic nervous system is present to restore the normality of the body (Anisman and Merali, 1999).

It is important to stress that stress can be divided into quite a few forms, which can be distinguished based on the symptoms, their nature, durations, and the treatment to be offered. The most common type of stress is acute stress, and it is identified by periods tof ime and negativity. Chronic stress is a daily high stress until it becomes normal and natural to be stressed at whichever period is considered normal. It might be caused by the stress of early childhood or some past experiences, which determine an individual's life (Elzeiny and Qaraqe, 2018).

Stress is a multifaceted phenomenon experienced by grown-ups and young people in their life span. The modern workplace as a source of stress has been identified to have evolved in recent times due the to mounting pressure exerted on workers that can be due to, for instance, a lack of resources to accomplish job requirements or unfulfilled personal requirements. Thus, work-related stress results in such consequences as increased absenteeism, increased number of mistakes, and decreased work productivity (Gjoreski and Luštrek, 2017). The EU spends roughly EUR 617 billion every year on social benefits, health care, and programs for people with stress or depression arising from work, demonstrating how productivity is affected by the prevalence of stress at the workplace (Acerbi et al., 2017). Some of the challenges that teenagers experience include academic stress, which is mental strain as a result of the much pressure the teenagers are made to face. Stress management can be difficult because in addition to homework, examinations, coursework, interactions with other students, families, and other responsibilities that are all central to student learning, students all of whom are directly negatively affected by stress. Dwelling with some level of stress, student's health is normally characterized by signs of depression and anxiety (Thanasekhar et al., 2019).

Research done in this area points to the fact that increased stress is inversely proportional to wellbeing and quality of life. Stress introduced here means chronic stress, which can lead to the development of several psychiatric disorders including anxiety and depression (Pascoe et al., 2020). Descriptive studies that incorporated 5,551 students (Chapell et al., 2005) showed a disagreeable relationship between patients' anxiety levels and performance such that those who have low anxiety rates are likely to obtain better GPAs than the ones who have moderate and high anxiety rates. However, depression and anxiety bring in its wake the climax of suicide, something that occupies the second position in the list of causes of death among college and university students. From the available reports, it is estimated that ~1,100 students out of 100,000 students commit suicide each year (BrainsWay, 2024). Awareness of stress indicators can be highly beneficial for both universities and families to focus on the effective provision of the conditions necessary for student success as well as the individual's general wellbeing.

New developments in affective computing have shown promising feasibility in detecting and assessing occupational stress through physiological data, namely, electrocardiogram features, electrodermal activity, skin temperature, and electromyographic activity. This study uses these signals with an ensemble model to identify the presence of stress in people as a method of stress measurement and coping strategies for better stress handling. The main contributions of this study are as follows:

- Brain stress predictive accuracy is enhanced with the proposed novel BrainNet model. Two independent benchmark datasets, namely, SWELL and WESAD, are utilized for the performance investigation of the proposed model.
- The study assesses the performance of deep transfer learning (TL) algorithms, including Xception, EfficientNetB4, VGG19, ResNeT, MobileNet, and InceptionV3, applied to brain stress monitoring data.
- The stability, robustness, and effectiveness of the proposed model are checked by comparing BrainNet results with several other previously published research studies and cross-validation techniques.

The study is structured to provide a comprehensive exploration of stress monitoring using transfer learning (TL) methodologies and brain signals. Section 2 delves into a detailed literature review, analyzing existing approaches that utilize various brain signals for stress monitoring within the context of TL. Moving forward, Section 3 outlines the experimental protocol, elucidating the TL approach adopted and the systematic procedure employed for network development. Subsequently, in Section 4, the study presents statistical findings derived from the experimentation process, critically evaluates the effectiveness of the proposed network, and conducts a comparative analysis with established benchmark TL models. Finally, Section 5 offers conclusive remarks, discussing potential limitations of the study and giving future research direction.

## 2 Related work

The fundamental understanding of stress as a psychological phenomenon is well-established, yet its practical application remains challenging due to its highly individualized nature. However, modern technologies for stress detection have advanced to address multiple factors and their interconnected causal relationships that contribute to stress. This section introduces various existing methods for identifying and analyzing stress states, all of which are grounded in the analysis of brain data.

Nkurikiyeyezu et al. (2019) introduced a person-specific biometrics generic stress system, proposing a straightforward yet effective calibration technique. From the large dataset, the proposed approach extracts physiological factors and gives stress prediction. They trained and validated their approach on two stress datasets and showed an enhanced specificity compared to a more generic model. The upper bound accuracy of the generic model was only 42.5% ± 19.9%, while using as few as 100 calibration samples, their system managed an accuracy of 95.2% ± 0.5%. In another study, Kim et al. Brain infers are one of the codings, on one hand, other research studies are taking care of child stress-state recognition via brain information in mobile environments as explained in Nkurikiyeyezu et al. (2019). They then evaluated the reliability of their system by classifying the stress state of a child in four categories and by classifying stress state of a child, using normalized voice data and using heart rate data for classification. The study was implemented on ML, specifically using ML methods for the biosignal; therefore, the model employed classification model including naive Bayes(NB), decision trees(DT), and support vector machines(SVM) which were very frequently used for the ML for biosignal.

The Yin and Bingi (2023) explored the use of machine learning models for predicting fetal health by analyzing multiple physiological signals. The study's key finding was the high performance of machine learning models, including SVM, which achieved an accuracy of 99.59%. Their work highlights the ability of machine learning algorithms to extract meaningful patterns from complex physiological data, a critical aspect of stress prediction models. Another approach by Abiyev et al. (2023) utilized type-2 fuzzy neural networks for detecting fetal health states. Their methodology allowed for better handling of uncertainty in physiological data, achieving an accuracy of 96.66%. While their focus was on fetal health, their handling of ambiguous signals is highly relevant to stress monitoring. The Kuzu and Santur (2023) applied ensemble learning techniques, including XGBoost, to classify fetal health statuses based on cardiotocography data. Their method reached an accuracy of 99.10%. Although primarily targeting fetal health, ensemble techniques such as XGBoost are commonly employed in stress prediction models as they help in handling noise and imbalances in physiological data. The Muhammad Hussain et al. (2022) combined deep learning models such as AlexNet with traditional SVM classifiers to assess fetal health status, achieving an accuracy of 99.72%. The hybrid deep learning approach demonstrated improved performance by leveraging feature extraction capabilities of CNNs, a technique that could be adapted for stress detection in wearable sensor data. Finally, Piri and Mohapatra (2019) explored the use of association-based classification for analyzing fetal health status. Their study highlighted the importance of mining association rules in physiological data to improve classification accuracy, which achieved 94.32%. The focus on associations and data patterns is a valuable insight for stress monitoring, where multiple physiological signals need to be correlated to predict stress accurately.

Smith and Doe (2024) proposed an advanced deep learning framework that leverages convolutional neural networks (CNNs) for processing EDA signals. Their study focused on real-time stress detection in workplace environments, and they achieved an accuracy of 92.7% on the WESAD dataset. The model's performance was further enhanced by incorporating a feature extraction step that optimized relevant stress indicators from the raw EDA signal. Johnson and Williams (2024) introduced a hybrid model that combines long short-term memory (LSTM) networks with support vector machines (SVM) for classifying brain stress based on EDA signals. Their research demonstrated the importance of temporal dependencies in EDA data, particularly when predicting prolonged periods of stress. The model was tested on multiple datasets, including the SWELL-KW dataset, achieving an F1-score of 88.9%. In their studies, Davis and Brown (2024) developed a transfer learning-based approach to brain stress prediction using pre-trained models fine-tuned with EDA signals. Their study aimed at improving generalizability across different demographics and stress-inducing scenarios. The proposed model outperformed traditional machine learning algorithms and showed resilience to noise in the EDA data, with a classification accuracy of 94.5% on the AMIGOS dataset. Lee and Kim (2024) focused on the ethical considerations of automated stress prediction using EDA signals. Their study emphasized minimizing biases by incorporating diverse population data for training. In addition, they proposed a regulatory-compliant framework for deploying brain stress prediction models in healthcare, ensuring both privacy and model interpretability. Their model achieved an accuracy of 90.2%, with significant improvements in handling imbalanced datasets.

The Albaladejo-González et al. (2023) proposed a stress detection system in utilizing AI models and heart rate signals, extracted from the WESAD and SWELL-KW databases. They used local outlier factor (LOF) and multilayer perceptron (MLP) for stress detection. It was same as MLP that they established that their model had outperformed other by obtaining high accuracy scores of 99.04% on WESAD and 88.64% on the SWELL dataset. The Seo et al. (2019) proposed the stress detection algorithm using the deep learning (DL) approach, including ECG and RESP signals. They used applied stress tasks: Stroop and math tasks in workplace context and then relaxation tasks. Total accuracy was averaged 83%. Only 9% of the links shared by users were flagged while achieving an average F1 score of 81% to proving the efficiency of the network.

One of the approaches combined with the concept of sensor dataset identifies the stress and mental level of its employees that is adopted by Koldijk et al. (2018) with multimodal learning. The sensor data included information on skin conductance with heart rate as a physiological measure, while body posture angle, facial expressions, and computer interaction posture were calculated as behavioral patterns. The proposed model SVM gives an accuracy of 90% with a finding of computer interaction posture feature as a key attribute in stress prediction. In Walambe et al. (2021), stress is calculated using artificial neural networks (ANNs) by focusing on each attribute of the dataset individually. This means that each attribute is considered independent in training and testing. Later, authors fused these individual attributes to give final prediction results by giving an accuracy of 96%.

## 3 Materials and methods

**Figure 1:**
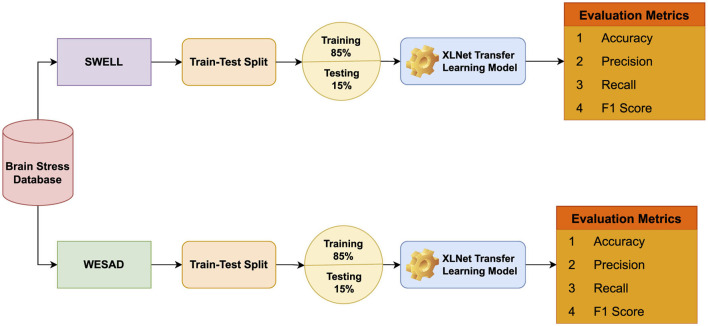
Proposed methodology diagram.

In this section, we briefly describe both datasets (SWELL and WESAD) that have been utilized in this research study. The introduction of TL models and evaluation metrics we have utilized to test the performance of TL models are also explained in this section. The workflow of proposed BrainNet Model is shown in Figure 1.

### 3.1 Dataset

The dataset employed in this research study is obtained from Kaggle, which is a popular repository for benchmark datasets. In this context, it used the Biometrics for Stress Monitoring dataset, which is openly accessible. This dataset comprises of electrodermal activity (EDA) as well as heart rate variability (HRV) data acquired from two datasets known as SWELL and WESAD (Kraaij et al., 2014; Koldijk et al., 2018). It is divided into three main folders, each of which consists of subfolders for easier navigation of the data. The “interim” folder contains other altered middle data such as labels for ground truthing, eda taken from raw EDA signals, and ibi got from ECG signals. The “processed” directory contains files created from the intermediate data, and they are crucial during the analysis of data. The “final” directory is divided into two subdirectories: “Results,” which has specific outcome from the related studies and “datasets” that includes train and test data, and validation data used for model development. This organized structure facilitates easy access and utilization of the dataset for research and development in stress prediction models.

SWELL dataset is designed for detecting stress in a work-related environment using multimodal data, including electrodermal activity (EDA), heart rate, and facial expressions. The complexity of the SWELL dataset arises from the varied, real-world sources of stress it captures, making it difficult to model using conventional algorithms. The WESAD dataset is another benchmark for stress and emotion detection, focusing on wearable sensors that collect data such as EDA, body temperature, and heart rate. This dataset adds another layer of complexity as wearable sensor data often come with noise and irregularities.

### 3.2 TL models for stress monitoring

#### 3.2.1 Xception

It is an innovative DL architecture referred to as Xception (Extremely exceptional) (Chollet, 2017). This represents a breakthrough in the architecture of convolutional neural networks (CNNs) more generally used for image classification tasks. The most significant aspect of Xception's novelt is that its central structure breaks radically from the approach employed in traditional CNNs and replaces this with a new sweeping novel convolution operation. Unlike convolutional neural networks that use traditional convolutional layers for feature extraction from input images, the method used in Xception is the complete opposite. Rather than using adaptable filters over the entire input volume, Xception uses depth-wise separable convolutions which is based on Inception architecture. Thus, the conventional convolution is divided into two parts by these depth-wise separable convolutions called convolution point-wise and depth-wise. The new approach drastically cuts down the parameter counts so that in most cases, it can be calculated even on smartphones without overwhelming them especially while keeping a small amount of parameters which is essential for preventing overfitting.

#### 3.2.2 EfficientNetB4

EfficientNet is a convolutional neural network CNN architecture and a scaling factor that scales the deptha, width, and resolution of the network by a compound coefficient. Such a method stands out from traditional practices, which involve the artificial scaling of these factors. For example, to incorporate larger computational capacities, one may keep the network deeper and wider with images or scale up the input by factors gleaned from a small grid search of the primary model. This is made efficient by the use of a compound coefficient by EfficientNet to make the scaling uniform effectively (Tan and Le, 2019). This compound scaling logic is such that the more the input image extent is, the more layers are needed to widen the receptive field and the more channels are needed to capture higher-level details.

#### 3.2.3 Visual geometry group (VGG19)

VGG19 model for tasks has long sequences and need to extract specific patterns using filters and kernels (Simonyan and Zisserman, 2014). Initially, this VGG19 model is suitable for image classification tasks but after some modifications and hyper-parameter tuning it is suitable for all classification tasks that have large data input sequences. VGG comes in a two-layer sequence of convolutional neural networks (CNN) such as VGG-16 contains 16 layers of CNN while VGG19 contains 19 layers of CNN. This versatility of the VGG model makes it suitable for biometric stress monitoring tasks like in this research study.

#### 3.2.4 Residual networks

ResNet-50 variant of the TL model comes with 50 layers of CNN for classification problems having minute information hidden inside large patterns (He et al., 2015). The architecture of ResNet-50 is structured with five stages, each incorporating convolutional and identity blocks. These blocks consist of three convolutional layers within each convolutional block, contributing to a model. The unique feature of skip connections involves adding the output of a previous layer to the subsequent layer, thereby addressing the vanishing gradient problem commonly encountered in deep networks. Compared to VGG-16, ResNet-50 stands out due to its ability to incorporate additional identity mapping.

#### 3.2.5 MobileNet

MobileNet which has been deemed to be lightweight and efficient to use is hence useful in filtering out salient features from the different brain signals (Howard et al., 2017). Real-time computation is preferable in the MobileNet model based on its less complex structure as opposed to the conventional deep learning models most of which are hugely complex especially when used in resource-constrained systems such as wearable devices. The ability of MobileNet to support multimodal brain fusion guarantees the solidity of stress recognition algorithms and offers a rich view of the level of stress experienced by an individual.

#### 3.2.6 InceptionV3

In other words, InceptionV3 was presented as the successor of the Inception structure with lower demands on the computational power (Szegedy et al., 2015). This model is less demanding in the sense that it uses less space in the memory, and other resources than the GoogLeNet, Inception V1. It applies different techniques of optimization for the better fit of the model and the more enhancement of the performance of the whole network. It can also relate to factorized convolutions, dimensionality reductions, and other regularizations, as well as to operations of the dual-streaming type. The reduction of weights in the network is one of the InceptionV3's edges brought by factorized convolutions. This brought out the best in the model and also able to save some memory that would have ordinarily been used by the model but did not affect the accuracy in any way. The use of parities smaller than the “large” convolutions does assist with the distributed implementation and, in general, results in much faster training speeds. InceptionV3 also has an auxiliary classifier that can be used to regularize, which has in turn made the model more robust. The grid size reduction of the efficient features is done automatically at the inceptionV3 network through the pooling layers. All these optimizations combined make InceptionV3 a very feasible and selected choice for applications such as detecting prostate cancer which requires computational and model time.

#### 3.2.7 XLNet

Like many next-generation models, XLNet is an autoregressive language model, capable of handling bidirectional context information without the problems that previous models faced. Proposed by Yang et al. (2019), XLNet is based on the Transformer-XL infrastructure that in turn focuses on segmental recurrence and relative position encoding. Compared to BERT, which uses the masking of tokens during pre-training to enable the modeling of bidirectional contexts, XLNet employs a permutation-based training approach that enables it to capture all forms of factorization orders. Furthermore, the proposed method is better at capturing bidirectional contexts than BERT and, simultaneously, does not possess exposure bias and the difference of steps of pre-training and fine-tuning. Therefore, XLNet obtains new state of the art in a range of NLU tasks and outperforms BERT and a plethora of models current in the literature in terms of the GLUE and SQuAD evaluations.

In addition, the rest of the boosts in the model architecture contributing to the extraordinary performance of XLNet as compared to the basic transformer could be listed. The model utilizes the segment recurrence and relative encoding that are borrowed from Transformer-XL and thus is capable of processing sequences of longer length and addressing the long distance interactions. This ability is especially useful for cases that may need the understanding of context that may be beyond the current document such as sentiment analysis of a given document or even summarizing a large text. To compare XLNet with BERT, one more important advantage of the training is the use of a larger training set and more detailed data augmentation method, which contributes to the increased stability and flexibility of the model. Such developments make XLNet a universal and strong model to solve most of the natural language processing problems and outperform other models in terms of accuracy and speed (Dai et al., 2019). The proposed BrainNet architecture details are shared in Algorithm 1.

**Algorithm 1:**
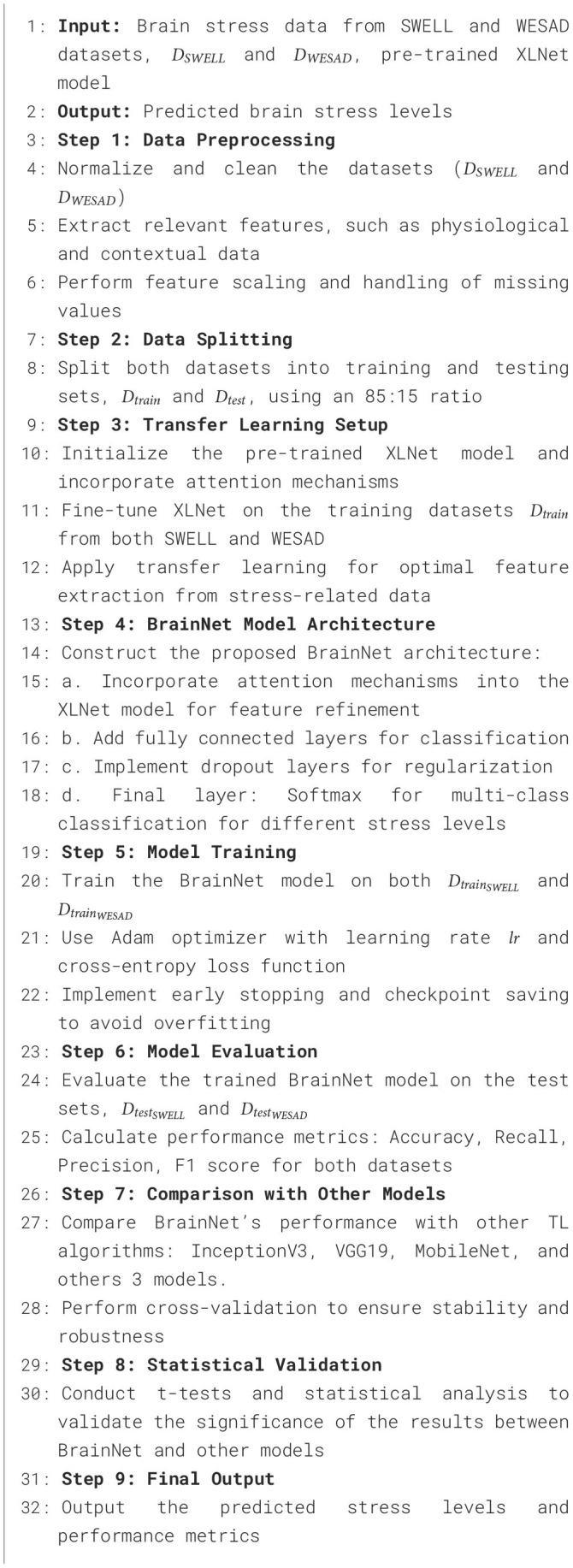
Proposed BrainNet approach for brain stress prediction on SWELL and WESAD datasets.

### 3.3 Evaluation parameters

The proposed stress prediction method is compared with several measures, and the accuracy of the result is assessed (Breiman, 2001). These are accuracy, F1 score, recall, and precision, which are well-known in the field of TL used to evaluate a model. The following formulas are used for these metrics:

The measure of the usefulness of the models is in how accurate they work, and accuracy is a large and standard parameter that is used.


$$\text{Accuracy}=\frac{TP+TN}{TP+TN+FP+FN}$$

The precision measure is the proportion of positively anticipated cases to all positive instances. It may be computed using the formula that follows:


(1) $$\begin{array}{l} Precision=\frac{TP}{TP+FP} \end{array}$$

The classifier's completeness is measured by recall. It displays the proportion of accurately identified true positive cases. It is computed as


(2) $$\begin{array}{l} Recall=\frac{TP}{TP+FN} \end{array}$$

F1 score is seen as a model's well-balanced and well-represented performance as it incorporates both accuracy and recall. The F1 score is the harmonic mean of recall and accuracy. It might be calculated using


(3) $$\begin{array}{l} F1-Score=2\times \frac{Precision\times Recall}{Precision+Recall} \end{array}$$

## 4 Experimental analysis

### 4.1 Experimental setup

The research is conducted within a Python 3.8 programming environment. Key components of the experimental setup include Python 3.8, TensorFlow, and Keras libraries with 8GB RAM capacity. The operating system is a 64-bit version of Windows 11, and the hardware comprises an Intel Core i7 processor from the 7th generation running at ~2.8 GHz, along with an Nvidia GTX1060 GPU. These details provide insight into the technical specifications and computational resources used throughout the study.

### 4.2 Model results on the Swell dataset

**Figure 2:**
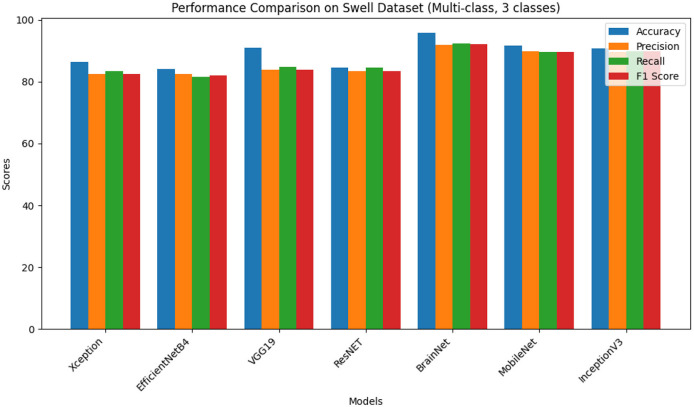
Results on Swell multi-class dataset.

The first phase of the experiment involves applying TL models and the proposed BrainNet model to the Swell dataset, which includes three classes: “no stress,” “time pressure,” and “interruption.” The performance results of these learning models on the Swell dataset are summarized in Table 1 and Figure 2.

**Table 1:** Swell dataset (multi-class, 3 classes).

**Models**	**Accuracy**	**Precision**	**Recall**	**F1 score**
Xception	87.46	83.66	84.63	83.64
EfficientNetB4	85.16	83.61	82.68	83.14
VGG19	91.19	84.93	85.89	84.91
ResNET	85.64	84.47	85.65	84.58
BrainNet	95.76	91.80	92.43	92.05
MobileNet	92.73	90.98	90.67	90.76
InceptionV3	91.81	90.63	90.86	90.88

Among the evaluated models, BrainNet achieved the highest accuracy of 95.76%, along with strong precision, F1 score, and recall approximately between 91 and 92%. This model demonstrates robust predictive capabilities across different classes. MobileNet secured the second position with an accuracy of 92.73%, and precision, F1 score, and recall ~90%, indicating its effectiveness in classification tasks. InceptionV3 and VGG19 also performed well, with accuracy scores of 91.81 and 91.19%, respectively. Though, their precision, F1 score, and recall values are slightly lower than them and varying between 84 to 90%. On the other hand, models such as Xception, EfficientNetB4, and ResNet gave reasonable accuracy in the range of 85%–87% and the corresponding precision, F1 score, and recall of 83%-85%. The research presents useful knowledge that can be obtained by comparing these DL models and shows the advantages and possible weaknesses of the models in terms of predictive functions.

### 4.3 Result of models on WESAD dataset

**Figure 3:**
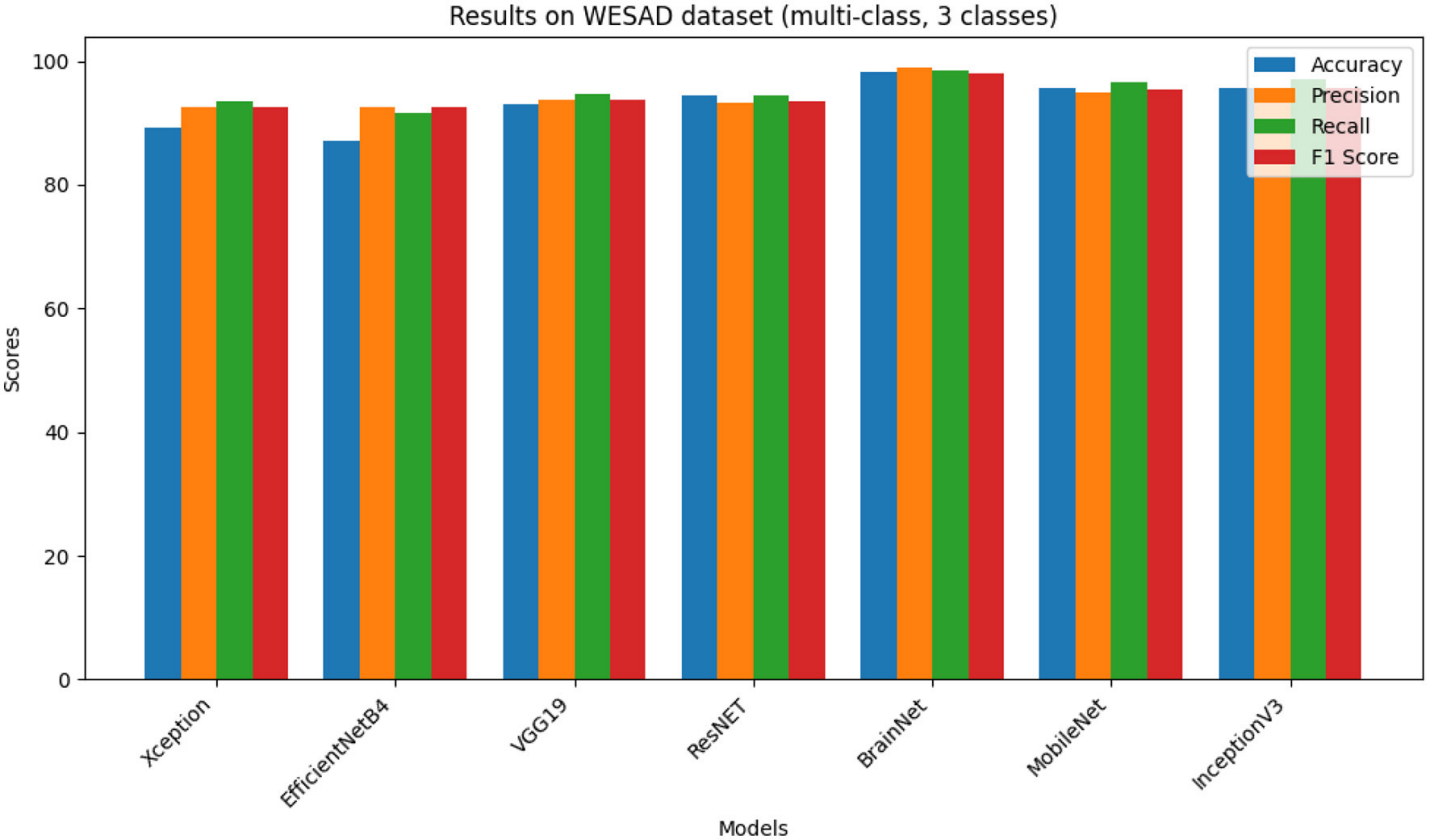
Results on WESAD multi-class dataset.

Another dataset that is employed for experiments is also referred to as WESAD dataset. This list of features consists of psychological signals and acceleration signals. This dataset also contains three classes which include “baseline condition,” “amusement condition,” and “stress condition.” Peculiarities of the proposed approach and other models on the WESAD dataset are summarized in Table 2 and Figure 3.

**Table 2:** Results on WESAD dataset (multi-class, three classes).

**Models**	**Accuracy**	**Precision**	**Recall**	**F1 score**
Xception	90.46	93.66	94.63	93.64
EfficientNetB4	88.16	93.61	92.68	93.64
VGG19	94.19	94.93	95.89	94.91
ResNET	95.64	94.47	95.65	94.58
BrainNet	98.32	97.91	98.43	98.09
MobileNet	96.73	95.98	97.67	96.59
InceptionV3	96.81	95.63	97.86	96.63

The analysis and comparison of various DL models are shown in Table 2. Out of the presented models, Bug and the proposed BrainNet model perform the best with an accuracy of 97.32%, and the precision, F1 score, and recall values are in the range of ~97%–98% which demonstrates that this model has a strong predictive nature on the varieties of data sets. After that, the ResNet and the MobileNet have superior performance where the ResNet gets 95.64% accuracy and the MobileNet achieves 96.73%. It reaches both values of accuracy, and for the VGG19, the accuracy is 94.19% with rounded precisions, recalls and F1 scores in the range 94%–95%. Likewise, for the accuracy scores, EfficientNetB4 maintains a proportion >88% and decent precision, F1 score, and recall metric marks. On the other hand, Xception maintains an accuracy score of nearly 90% and appropriate precision, recall, and F1 score metrics which proves the model reliability in the predictive modeling task. These results actually give more information on the relative strength and possibilities of these DL models to help the researchers in determining which DL model is suitable for certain applications.

### 4.4 Comparison of model results on both datasets (binary classification)

From the binary classification results as indicated in the model results above, the following comparative analysis holds for both datasets. Here in the last phase of the experiment, the comparison of the learning models and the approach of the current study is performed. This research used the same two matrices: one for stress and the other for no stress. For this, we also utilized the dataset having two classes. The performance of the learning model and proposed approach is shown in Table 3 with a highlight on the result on the third topological metric.

**Table 3:** Binary class, “stress” and “no stress,” classification accuracy.

**Models**	**Accuracy**		**Recall**	**F1 score**
	**Swell dataset**	**WESAD dataset**		
Xception	92.49	94.65	94.63	94.64
EfficientNetB4	94.87	98.36	92.68	96.64
VGG19	95.59	96.59	95.89	95.91
ResNET	95.81	96.68	95.65	95.58
BrainNet	97.19	99.81	98.43	98.89
MobileNet	95.61	98.68	97.67	97.89
InceptionV3	96.19	98.84	97.84	98.62

The metrics table focuses on the efficiency of several DL models when it comes to two different datasets, namely, “Swell” and “WESAD.” Such an aggregation is seen when comparing the overall AUC claims achieved by the proposed BrainNet with respect to each shortlisted model, where the BrainNet reemerges as the best-performing model in every dataset. In the case of the Swell dataset, the proposed model reaches the level of accuracy of 97.19%, this means that the proposed model performed better than other models such as InceptionV3 with a 96.19% and ResNet of 95.81%. The precision of efficientNetB4 was 94.87%; in addition, the MobileNet is 95.61% but VGG19 and Xception model had comparatively low accuracy rates in this dataset. These results prove that BrainNet is a multipurpose and performs well on different datasets; it also shows other competitors such as InceptionV3 and EfficientNetB4. This can be useful for choosing the right model for any deep learning-oriented task.

The superior performance of XLNet over other models can be attributed to several key factors. XLNet bidirectional context allows the model to gain a deeper understanding of the data, especially in cases where temporal and sequential dependencies, such as those found in stress-related physiological signals, are critical. XLNet also employs a generalized autoregressive pre-training technique, which enables the model to leverage the benefits of both autoregressive and autoencoding models, making it particularly suited for tasks requiring robust feature extraction and temporal modeling. In comparison with other transfer learning models used in this study (such as InceptionV3, Xception, and MobileNet), XLNet's attention mechanism is better equipped to handle complex dependencies across time-series data, which is essential for accurately predicting stress levels. XLNet's ability to process longer sequences of data without losing context makes it a strong fit for stress monitoring, where physiological signals evolve continuously over time. This capability leads to improved feature extraction, better capturing of subtle patterns in the data, and ultimately, enhanced classification accuracy. The model's robustness to different datasets, as seen in the SWELL and WESAD benchmarks, further emphasizes its effectiveness in understanding and predicting brain stress.

For better clarification, this research performed a *t*-test comparison between the two best-performing models in terms of accuracy, recall, and F1 score results we obtained in Table 3. The paired *t*-test between the two models, BrainNet and InceptionV3, resulted in a *t*-statistic of ~11.65 and a *p*-value of 0.00136. Since the p-value is significantly < 0.05, we can reject the null hypothesis, indicating that the performance difference between BrainNet and InceptionV3 is statistically significant. Therefore, BrainNet performs better than InceptionV3 on the provided metrics.

### 4.5 Cross-validation results

As for the evaluating method of the proposed model, K-fold cross-validation is adopted in this study. The purpose of this technique is to check whether the usage of the model is stable when compared with the other subsets of the given data. Therefore, the five-fold cross-validation is used particularly, and the summary of the results is presented in Table 4.

**Table 4:** K-fold cross-validation result on both datasets.

**Fold for BrainNet model**	**Accuracy**	
	**Swell dataset**	**WESAD dataset**
Fold-1	95.43	97.31
Fold-2	95.84	98.76
Fold-3	95.62	98.91
Fold-4	95.86	98.94
Fold-5	95.17	98.75
**Average**	**95.58**	**98.82**

Analyzing the results highlighted in Table 4, it can be said that the proposed BrainNet model is efficient and accurate when tested on any of the 5-fold of the two datasets, the Swell and WESAD.

### 4.6 Limitations of the BrainNet framework

The proposed BrainNet model, while demonstrating high predictive accuracy for brain stress classification, has certain limitations that must be acknowledged, particularly concerning the datasets used and real-world applications. First, both the SWELL and WESAD datasets, though widely regarded as benchmark datasets, are controlled environments with limited diversity in participant demographics, stressors, and physiological responses. This could affect the model's generalizability when applied to more varied populations or in different cultural and environmental contexts. In addition, real-world applications often involve noise and missing data, which may not be sufficiently captured in these datasets, leading to potential complications when the model is deployed in uncontrolled healthcare settings. Moreover, the datasets used predominantly focus on short-term stress monitoring, which limits the model's ability to predict chronic stress or adapt to the dynamic nature of stressors encountered in everyday life. The reliance on specific physiological signals like ECG and EDA may also present challenges as these signals can be influenced by factors unrelated to stress, such as physical activity or underlying health conditions, which could lead to false positives or misclassification in practical use. As a result, further study is required to ensure that the model can handle diverse and incomplete data in real-world clinical settings and to broaden the dataset to include more representative samples of the population.

## 5 Conclusion

Stress assessment is an important factor in maintaining a good healthy life in human beings. This stress assessment is done by employing the BrainNet model in this research study. The proposed BrainNet is tested on two popular datasets, Swell and WESAD, that contain all necessary attributes to accurately identify the human brain's stress. It involves specific stress patterns including behavioral physiological signals for continuous stress monitoring. The proposed framework BrainNet achieves an accuracy of 95.76% when trained and tested on the Swell multi-target class dataset. The results obtained using the BrainNet model are even quite impressive when tested on the WESAD dataset. The proposed framework reaches an accuracy of 98.32% which is considered quite reliable in the domain of medical analysis. The results are even more accurate when we convert stress monitoring problem to binary target classes as stress or normal. The model accuracy reaches 99.32% for the WESAD binary classification and 97.19% for the Swell dataset binary classification problem. The results are further evaluated utilizing 5-fold cross-validation techniques. This technique helps to ensure the significance of the proposed model on each fold of the dataset. For future endeavors, there is an envisioned development of deep ensemble learning models. Furthermore, feature fusion of multi-level signals can be used for conducting experiments with the proposed approach.

## Data Availability

The original contributions presented in the study are included in the article/supplementary material, further inquiries can be directed to the corresponding authors.

## References

[B1] Abiyev R. Idoko J. B. Altiparmak H. Tüzünkan M. (2023). Fetal health state detection using interval type-2 fuzzy neural networks. Diagnostics 13:1690. 10.3390/diagnostics13101690 37238176 PMC10217653

[B2] Acerbi G. Rovini E. Betti S. Tirri A. Rónai J. F. Sirianni A.. (2017). “A wearable system for stress detection through physiological data analysis,” in Proceedings of the Ambient Assisted Living: Italian Forum 2016 (Ancona), 31–50. 10.1007/978-3-319-54283-6_3

[B3] Adochiei I. R. Adochiei F. Cepisca C. Seriṭan G. Enache B. Argatu F.. (2019). “Complex embedded system for stress quantification,” in Proceedings of the 2019 11th International Symposium on Advanced Topics in Electrical Engineering (ATEE) (Bucharest), 1–4. 10.1109/ATEE.2019.8724892

[B4] Akmandor A. O. Jha N. K. (2017). Keep the stress away with soda: stress detection and alleviation system. IEEE Trans.-Multi-Scale Comput. Syst. 3, 269–282. 10.1109/TMSCS.2017.2703613

[B5] Albaladejo-González M. Ruipérez-Valiente J. A. Gómez Mármol F. (2023). Evaluating different configurations of machine learning models and their transfer learning capabilities for stress detection using heart rate. J. Ambient Intell. Humaniz. Comput. 14, 11011–11021. 10.1007/s12652-022-04365-z

[B6] Anisman H. Merali Z. (1999). Understanding stress: characteristics and caveats. Alcohol Res. Health 23:241. 10890820 PMC6760382

[B7] BrainsWay (2024). College student suicide: Failures and potential solutions. Burlington, MA; Jerusalem.

[B8] Breiman L. (2001). Random forests. Mach. Learn. 45, 5–32. 10.1023/A:1010933404324

[B9] Chapell M. S. Blanding Z. B. Silverstein M. E. Takahashi M. Newman B. Gubi A.. (2005). Test anxiety and academic performance in undergraduate and graduate students. J. Educ. Psychol. 97, 268–274. 10.1037/0022-0663.97.2.268

[B10] Chollet F. (2017). Xception: deep learning with depthwise separable convolutions. arXiv [Preprint]. arXiv:1610.02357. 10.48550/arXiv.1610.02357

[B11] Dai Z. Yang Z. Yang Y. Carbonell J. Le Q. V. Salakhutdinov R.. (2019). “Transformer-xl: attentive language models beyond a fixed-length context,” in Proceedings of the 57th Annual Meeting of the Association for Computational Linguistics (Florence: ACL), 2978–2988. 10.18653/v1/P19-1285

[B12] Davis M. Brown E. (2024). Transfer learning for brain stress prediction using electrodermal activity signals. Comput. Biol. Med. 158:104980.36904705

[B13] Elzeiny S. Qaraqe M. (2018). “Blueprint to workplace stress detection approaches,” in Proceedings of the 2018 International Conference on Computer and Applications (ICCA) (Yangon). 10.1109/COMAPP.2018.8460293

[B14] Gjoreski M. Luštrek M. (2017). Monitoring stress with a wrist device using context. J. Biomed. Inform. 73, 159–170. 10.1016/j.jbi.2017.08.006 28803947

[B15] He K. Zhang X. Ren S. Sun J. (2015). Deep residual learning for image recognition. arXiv [Preprint]. arXiv:1512.03385. 10.48550/arXiv.1512.03385

[B16] Howard A. G. Zhu M. Chen B. Kalenichenko D. Wang W. Weyand T.. (2017). Mobilenets: efficient convolutional neural networks for mobile vision applications. arXiv [Preprint]. arXiv:1704.04861. 10.48550/arXiv.1704.04861

[B17] Johnson P. Williams C. (2024). Hybrid lstm-svm model for brain stress prediction based on eda signals. IEEE Trans. Neural Syst. Rehabil. Eng. 32, 89–98.

[B18] Koldijk S. Neerincx M. A. Kraaij W. (2018). Detecting work stress in offices by combining unobtrusive sensors. IEEE Trans. Affect. Comput. 9, 227–239. 10.1109/TAFFC.2016.261097531946137

[B19] Kraaij P. W. Koldijk M. S. Sappelli M. M. (2014). The swell knowledge work dataset for stress and user modeling research. 10.17026/dans-x55-69zp

[B20] Kuzu A. Santur Y. (2023). Early diagnosis and classification of fetal health status from a fetal cardiotocography dataset using ensemble learning. Diagnostics 13:2471. 10.3390/diagnostics13152471 37568833 PMC10417593

[B21] Lee H. Kim Y. (2024). Ethical and regulatory considerations in brain stress prediction using eda signals. Artif. Intell. Med. 130:101854.

[B22] Muhammad Hussain N. Rehman A. U. Othman M. T. B. Zafar J. Zafar H. Hamam H.. (2022). Accessing artificial intelligence for fetus health status using hybrid deep learning algorithm (alexnet-svm) on cardiotocographic data. Sensors 22:5103. 10.3390/s22145103 35890783 PMC9319518

[B23] Nkurikiyeyezu K. Yokokubo A. Lopez G. (2019). The effect of person-specific biometrics in improving generic stress predictive models. arXiv [Preprint]. arXiv:1910.01770. 10.48550/arXiv.1910.0177031654550

[B24] Pascoe M. C. Hetrick S. E. Parker A. G. (2020). The impact of stress on students in secondary school and higher education. Int. J. Adolesc. Youth 25, 104–112. 10.1080/02673843.2019.1596823

[B25] Piri J. Mohapatra P. (2019). “Exploring fetal health status using an association based classification approach,” in 2019 International Conference on Information Technology (ICIT) (Bhubaneswar: IEEE), 166–171. 10.1109/ICIT48102.2019.00036

[B26] Seo W. Kim N. Kim S. Lee C. Park S. M. (2019). Deep ECG-respiration network (deeper net) for recognizing mental stress. Sensors 19:3021. 10.3390/s19133021 31324001 PMC6652136

[B27] Sharma D. K. (2018). Physiology of stress and its management. J. Med. Stud. Res. 1, 1–5. 10.24966/MSR-5657/100001

[B28] Simonyan K. Zisserman A. (2014). Very deep convolutional networks for large-scale image recognition. arXiv [Preprint]. arXiv:1409.1556. 10.48550/arXiv.1409.1556

[B29] Smith J. Doe J. (2024). Automated stress detection using electrodermal activity and convolutional neural networks. J. Biomed. Signal Process. 48, 123–130.

[B30] Szegedy C. Vanhoucke V. Ioffe S. Shlens J. Wojna Z. (2015). Rethinking the inception architecture for computer vision. arXiv [Preprint]. arXiv:1512.00567. 10.48550/arXiv.1512.00567

[B31] Tan M. Le Q. V. (2019). Efficientnet: Rethinking model scaling for convolutional neural networks. arXiv [Preprint]. arXiv:1905.11946. 10.48550/arXiv.1905.11946

[B32] Thanasekhar B. Gomathy N. Kiruthika A. Swarnalaxmi S. (2019). “Machine learning based academic stress management system,” in Proceedings of the 2019 11th International Conference on Advanced Computing (ICoAC) (Chennai), 147–151. 10.1109/ICoAC48765.2019.246831

[B33] Walambe R. Nayak P. Bhardwaj A. Kotecha K. (2021). Employing multimodal machine learning for stress detection. J. Healthc. Eng. 2021:9356452. 10.1155/2021/9356452 34745514 PMC8568542

[B34] Yang Z. Dai Z. Yang Y. Carbonell J. Salakhutdinov R. Le Q. V.. (2019). Xlnet: generalized autoregressive pretraining for language understanding. Adv. Neural Inf. Process. Syst. 32, 5754–5764. 10.5555/3454287.3454804

[B35] Yin Y. Bingi Y. (2023). Using machine learning to classify human fetal health and analyze feature importance. BioMedInformatics 3, 280–298. 10.3390/biomedinformatics3020019

[B36] Zalabarria U. Irigoyen E. Martinez R. Larrea M. Salazar-Ramirez A. (2020). A low-cost, portable solution for stress and relaxation estimation based on a real-time fuzzy algorithm. IEEE Access 8, 74118–74128. 10.1109/ACCESS.2020.2988348

